# 

**Published:** 1897-01

**Authors:** 


					﻿• Vol. XVII.
JANUARY, 1897.
NO. 1.
THE
HOMEOPATHIC PHYSICIAN,
A Monthly Journal of Homoeopathic Materia Medica
and Clinical Medicine.
** He it a freeman whom truth makes free, and all are slaves beside.". -
"Seek the Truth: come whence it may, cost what it will."
BDITED AND PUBLISHED BT
WALTER M. JAMES, M. D.
PHILADELPHIA
ZsTo. 1231 LOCUST STIEfcZEZET.
LONDON:
ALFRED HEATH & CO., 8ole Agents for Great Britain,
114 Ebury 8treet, Eaton 8quare,
■Yearly Subscription, $2.SO, invariably in advance. Single numbers, 25 cts.
Entered nt tbe Philadelphia Post-Office as Second-Class Mail Matter.
				

